# Stigma matters in ending tuberculosis: Nationwide survey of stigma in Ethiopia

**DOI:** 10.1186/s12889-019-7915-6

**Published:** 2020-02-06

**Authors:** Daniel G. Datiko, Degu Jerene, Pedro Suarez

**Affiliations:** 1 Challenge TB/Ethiopia and Management Sciences for Health/Ethiopia, P.O. Box 1157, Code 1250 Addis Ababa, Ethiopia; 2 Management Sciences for Health, Senior Director Infectious Diseases Cluster, Arlington, USA

**Keywords:** Tuberculosis, Stigma, Ethiopia

## Abstract

**Background:**

Tuberculosis (TB) affects, and claims the lives of, millions every year. Despite efforts to find and treat TB, about four million cases were missed globally in 2017. Barriers to accessing health care, inadequate health-seeking behavior of the community, poor socioeconomic conditions, and stigma are major determinants of this gap. Unfortunately, TB-related stigma remains unexplored in Ethiopia.

**Methods:**

This mixed methods survey was conducted using multistage cluster sampling to identify 32 districts and 8 sub-cities, from which 40 health centers were randomly selected. Twenty-one TB patients and 21 family members were enrolled from each health center, and 11 household members from each community in the catchment population.

**Results:**

A total of 3463 participants (844 TB patients, 836 from their families, and 1783 from the general population) were enrolled for the study. The mean age and standard deviation were 34.3 ± 12.9 years for both sexes (34.9 ± 13.2 for men and 33.8 ± 12.5 for women). Fifty percent of the study participants were women; 32.1% were illiterate; and 19.8% came from the lowest wealth quintile. The mean stigma score was 18.6 for the general population, 20.5 for families, and 21.3 for TB patients. The general population of Addis Ababa (AOR: 0.1 [95% CI: 0.06–0.17]), those educated above secondary school (AOR: 0.58 [95% CI: 0.39–0.87]), and those with a high score for knowledge about TB (AOR: 0.62 [95% CI: 0.49–0.78]) had low stigma scores. Families of TB patients who attended above secondary school (AOR: 0.37 [95% CI: 0.23–0.61]) had low stigma scores. TB patients educated above secondary school (AOR: 0.61 [95% CI: 0.38–0.97]) had lower stigma scores, while those in the first (AOR: 1.93: 95% CI 1.05–3.57) and third quintiles (AOR: 1.81: 95% CI: 1.08–3.05) had stigma scores twice as high as those in the highest quintile. Fear of job loss (32.5%), isolation (15.3%), and feeling avoided (9.3%) affected disclosure about TB.

**Conclusions:**

More than a third of Ethiopians have high scores for TB-related stigma, which were associated with educational status, poverty, and lack of awareness about TB. Stigma matters in TB prevention, care, and treatment and warrants stigma reduction interventions.

## Background

Tuberculosis (TB) is a worldwide public health crisis. A third of the world’s population is infected, and 10 million people developed TB disease, while 1.6 million died of TB, in 2017. National TB Programs (NTPs) have diagnosed and treated millions of TB cases over the years. However, about 4 million TB cases were missed globally in 2017 alone [1]. This gap could be due to problems related to health service delivery and poor health-seeking behavior of the community, which are affected by factors that include awareness about TB, gender, stigma, and constrained socioeconomic conditions [2–4].

Community health-seeking behavior is also affected by the accessibility of services, the availability and quality of services, and cultural factors. These vary by the type of health condition and the views and perceptions of the clients and communities, which are expressed in a range of behaviors, including stigma [4]. Stigma is described as social determinant of health because it is shaped by community norms, interpersonal relations, and health institutions’ culture [5, 6]. Unfortunately, people with living some diseases, including TB, HIV, and leprosy, face deep-rooted and longstanding stigma within the community [7, 8].

Understanding of stigma toward TB, unlike HIV, is limited [9]. TB is primarily a disease of the poor and is associated with high stigma in the community. In addition to the stigmatizing community response [10, 11], self-stigmatization by TB patients affects the will of TB patients to seek care and adhere to their treatment [12], which is also affected by the association of TB with HIV and negative cultural beliefs or norms [13]. Moreover, victim blaming by health workers and the community, such as associating TB with bad behavior on the part of patients, reinforces the grip of stigma in communities and the health system [14].

Studies have shown higher stigma among the poor, the less educated, women, and socially disadvantaged communities. However, the magnitude of stigma varies across settings and is worst when a person is discriminated against by society and household members, with negative consequences on marital life, quality of life, and future opportunities [15, 16].

Understanding perceptions about TB and community misconceptions is an entry to designing patient-centered services. Qualitative studies have reported that patients prefer to be treated well and receive adequate counseling at appropriate times, and want supportive mechanisms during their illness [17, 18]. But negative perceptions about TB affect patients’ capacity to disclose their disease status to relatives and family members. This has demoralized patients and resulted in selective disclosure of their status only to trusted community members [19]. However, in communities with better awareness about TB, where health care workers are supportive and adequate patient support mechanisms are available, the magnitude of stigma was reported to be low [10].

Ethiopia has a high burden of TB, TB/HIV, and multidrug-resistant TB, with comorbidities and high stigma [8, 9, 20]. Nearly 30% of estimated TB cases are still missed by the NTP. High stigma among the poor, women, and rural communities could be one of the factors contributing to the case detection gap [21–23]. However, there has been no large-scale study of TB-related stigma. Therefore, we aim to describe the magnitude of stigma related to TB in communities: TB patients, their families, and the general population of Ethiopia.

## Methods

### Study setting and population

This is the first national stigma survey conducted in seven regions and two city administrations of Ethiopia. The Challenge TB project operates in nine regions, covering 92% of the national population. The NTP started implementing the WHO-recommended DOTS strategy in 1995. Currently, 256 hospitals and 3390 health centers provide TB services, and over 16,000 health posts deliver community-based TB care. The community health services mainly focus on conducting regular health education sessions, identifying and referring presumptive TB cases, and providing adherence support. Despite these efforts, the NTP continues to miss a third of estimated cases [1].

### Study design and sampling

This is a mixed methods study conducted from October to November 2017 in the nine Challenge TB–supported regions of Ethiopia.

### Sample size for the quantitative study

A single population proportion formula was used to estimate sample size [24]. A design effect of 2 and 10% was added to compensate for the non-response rate. A total of 3463 participants were enrolled for the study: 1783 from the general population, 836 from families of TB patients, and 844 TB patients.

### Study area

Ethiopia is administratively divided into nine regional states and two city administrations. This survey was conducted in seven regions and two city administrations. From these, 16 zones (provinces) and four sub-cities were selected. From each zone or sub-city, two districts were randomly selected. From each district, one rural and one urban *kebele* (lowest administrative unit with an average population of five thousands) were identified for the study. Finally, from the total of 40 districts selected, 80 kebeles were included in the study. The kebeles were divided into *gots* or clusters as the final study unit. Households were identified by systematic sampling, and 22 household participants were enrolled from each *got* or cluster.

Forty health centers were identified to enroll TB patients and their families. From each health center, we interviewed 11 TB patients and their families after obtaining informed consent. We recruited TB patients who were at least 18 years old who had been on treatment for at least 1 month. From their households, a household member who was at least 18 years old who had lived in the house for at least 6 months was selected by lottery.

### Qualitative studies

We conducted 18 focus group discussions (FGDs) and 76 in-depth interviews (IDIs). The kebele administrators assisted the research team in the identification of participants. The participants in the IDIs were selected from program managers (10 regional, 8 provincial, and 10 district TB focal persons), 12 health care workers, 12 TB patients, 12 family members of TB patients, and 12 health extension workers (HEWs), who are community health workers. TB patients and families of TB patients were approached in the health centers, while participants of FGD from the community were identified by kebele administrators.

### Data collection

To collect quantitative data, we pre-tested and used semistructured questionnaires from the WHO guide, which contains a generic questionnaire for data collection about stigma and wealth index [(2)]. The questionnaire has variables related to perception of TB patients toward themselves, anticipated stigma from their households and the community, and enacted stigma that the patients had experienced.

Trained data collectors were employed for the study. Tablets for data collection used web-based CSPro software Version 7.2 (Census and Survey Processing System, US Census Bureau and ICF Macro; 2019).

FGDs and IDIs were conducted in the local languages by trained, experienced data collectors using pretested, semistructured, and open-ended topic guides. The FGDS and IDIs were audiotaped. Supervisors were assigned to conduct random data checks and household visits. Built-in validation checks (character, data type, range, limit, required fields, skip, etc.) were designed in the questionnaire, and a regular check was done by a central CSPro expert, the data manager.

### Data analysis

Quantitative data were extracted from the web-based system and exported to IBM SPSS Statistics Version 25 (Armonk, NY, USA: IBM; 2019) for analysis. Descriptive analysis was done using SPSS. Binary logistic and multivariate logistic regression, for variables with *p* < 0.25, were used.

Knowledge scores were constructed using the total number of interview questions employed to assess the knowledge of the study participants and the total number of expected correct answers. We calculated knowledge scores using the mean of the number of correct answers given by the study participants as a cut-off point to categorize the knowledge scores into high or low. The study participants who answered above the mean score were classified as having a high knowledge score, while those who answered below the mean score were classified as having a low knowledge score.

Wealth-related variables were initially constructed for rural and urban populations, and later a common wealth index was constructed using variables that were considered common both rural and urban areas. Finally, both the rural and urban wealth index regression coefficients were mapped into the common wealth index, resulting in a composite “national” wealth index, which was categorized into quintiles.

Qualitative data were imported to OpenCode software (version 3.6) and analyzed using thematic content analysis. Direct verbatim quotations and results from the coding and categorization were used to develop the narrative.

### Ethics approval and consent to participate

Ethical clearance was obtained from the Ethics Review Board of the Ministry of Science and Technology. We obtained support letters from the Federal Ministry of Health. We also sought and received informed verbal consent from the study participants. The Ethics Review Board approved informed verbal consent for the study.

## Results

### Sociodemographic and economic characteristics

#### General population

Among the 1783 study participants interviewed, the mean age (SD) was 34.6 years (SD ± 12.9). Of the total number of participants, 828 (46.8%) were men, 48.7% of whom were heads of household, and 33.5% of whom were married. Among the study participants from general population, 66% were married, 22% were housewives, and 30.1% of the study participants could not read and write (Table 1).

**Table 1:** Sociodemographic characteristics of the study population in Ethiopia

		General population		Families of TB patients		TB patients	
Variables	Categories	Number	%	Number	%	Number	%
Gender	Male	835	46.8	407	48.7	488	57.8
	Female	948	53.2	429	51.3	356	42.2
Age in years	18–30	872	48.9	405	48.4	251	29.7
	31–60	828	46.4	414	49.5	246	29.1
	>60	83	4.7	17	2.0	164	19.4
Relationship	Head	869	48.7	329	39.4	93	11.0
	Spouse	597	33.5	248	29.7	52	6.2
	Son/Daughter	266	14.9	160	19.1	38	4.5
	Other relative	44	2.5	88	10.5	396	46.9
	Non-relative	7	0.4	11	1.3	189	22.4
Marital Status	Married	1176	66	543	65.0	186	22.0
	Never married	343	19.2	203	24.3	7	0.8
	Divorced	143	8	46	5.5	4	0.5
	Widowed	107	6	38	4.5	434	51.4
	Living together	14	0.8	6	0.7	283	33.5
Educational Status	Illiterate	537	30.1	283	33.9	66	7.8
	Read and write only	128	7.2	51	6.1	57	6.8
	Primary	517	29	227	27.2	4	0.5
	Secondary	398	22.3	173	20.7	292	34.7
	Above secondary	203	11.4	102	12.2	40	4.7
Occupation	Employed	210	11.8	126	15.1	263	31.2
	House wife	393	22	178	21.3	163	19.3
	Farmer	322	18.1	178	21.3	85	10.1
	Daily laborer	179	10	80	9.6	102	12.1
	Trader	381	21.4	121	14.5	114	13.5
	Student	136	7.6	84	10.0	203	24.1
	No job/dependent	114	6.4	47	5.6	103	12.2
	House maid	38	2.1	19	2.3	85	10.1
	Other	10	0.6	3	0.4	109	12.9
Wealth Quintile	Lowest	355	19.9	166	19.9	102	12.1
	Second	350	19.6	169	20.2	18	2.1
	Third	365	20.5	162	19.4	8	0.9
	Fourth	352	19.7	173	20.7	168	19.9
	Highest	361	20.2	166	19.9	169	20.0

#### Families of TB patients

Of the 836 family members of TB patients interviewed, the mean (SD) age was 33.8 (11.6). In this group, 48.7% were males, 39.4% were head of households, 65% were married, 33.9% were illiterate, and 21.3% were farmers (Table 1).

#### TB patients

Of the 844 TB patients interviewed, the mean (SD) age was 34 (13.8) years, and 29.7% were in the age range 18–30 years. More than half (57.8%) were males, 46.9% were heads of household, 51.4% were married, 24.1% were farmers, and 34.7% could not read and write (Table 1). The mean family size and number of people sleeping per room were 4.5 and 3.6, respectively. Among the TB patients, more than a third (37.4%) had at least one child under the age of five.

### Attitudes about and stigma toward tuberculosis

#### General population

Almost two-thirds (64.5%) of the study participants reported that they could cope with TB, but 31.9% expressed fear. When asked who they would tell, 82.7% of participants reported that they would inform a doctor, while 21.5% would tell a spouse, 16.5% close friends, and 25.7% family members. The majority (95.8%) of the participants reported that they would go to public facilities, while 14% reported that they would go to private facilities. More than two-thirds (68.5%) of the community felt compassion for TB patients, but 20.5% stated that TB patients are rejected by the community (Table 2).

**Table 2:** Attitude and stigma towards tuberculosis among the general population

Variables		#	%
Do you think you can get TB (*n* = 1668)		756	45.3
Reaction if you were found out that you have TB (*N* = 1668)	Cope with it	1076	64.5
	Fear	532	31.9
	Surprise	80	4.8
	Shame	55	3.3
	Embarrassment	33	2
	Sadness or hopelessness	78	4.7
	Other	9	0.5
Who would you talk to about your illness if you had TB? (*N* = 1668)	Doctor/other medical worker	1380	82.7
	Spouse	358	21.5
	Parent	450	27
	Children	169	10.1
	Other family member	428	25.7
	Close friend	276	16.5
	No one	18	1.1
	Others	5	0.03
What would you do if you thought you had symptoms of TB?	Go to public health facility	1598	95.8
	Go to private health facility	233	14
	Go to pharmacy	46	2.8
	Go to spiritual/traditional healer	25	1.5
	Pursue other self-treatment options	4	0.2
	Others	2	0.1
	Don’t know	11	0.7
If you would not go to health facility, what is the reason? (*N* = 24)	Not sure where to go	7	29.2
	Cost	11	45.8
	Transportation related	2	8.3
	Don’t trust health workers	4	16.7
	Would be cured by religion	1	0.1
If you had symptoms of TB, at what point would you go to the health facility?	Immediately	1080	64.7
	In few days	305	18.3
	One to 2 weeks	172	10.3
	After 2 weeks	93	5.6
	I will not go to health facility	14	0.8
	Other	4	0.2
How expensive do you think TB diagnosis and treatment in this country?	It is free of charge	782	46.9
	It is reasonably priced	187	11.2
	It is moderately expensive	139	8.3
	It is very expensive	142	8.5
	Don’t know	418	25.1
Know people who have/had TB		815	48.9
Statement closest to your feeling about people with TB	I feel compassion and desire to help	1142	68.5
	I feel compassion but tend to stay away from these people	208	12.5
	It is their problem and I don’t want to get TB by trying to help them	74	4.4
	I fear them because they may infect me	98	5.9
	I have no particular feeling	145	8.7
	Other	1	0.1
How TB patient usually regarded/treated In your community?	Most people reject him/her	342	20.5
	Most people are friendly, but they generally try to avoid	295	17.7
	The community mostly supports and helps him/her	683	40.9
	I don’t have the experience	345	20.7
	Others	3	0.2

Some participants (11.9%) would keep TB disease secret, but 84.5% would disclose their status, and if they did so, 84.6% would tell family members. Of the participants, 18.1% reported that the community would think less of them, 24.2% said the community would avoid them, 15.1% said they would be asked to stay away, and 14.9% would be ashamed. Of the general population, 9.8% responded that they would not disclose TB disease to a confidant; 16.4% would think less of themselves, and 6.5% expected that family would think less of them (Table 3).

**Table 3:** Response of the general population to TB stigma related questions

Stigma related questions (*N* = 1668)	Agree		Indifferent		Disagree	
	#	%	#	%	#	%
If yourself got TB, you would want it to remain secret.	198	11.9	61	3.7	1409	84.5
If a member of your family got TB, you would want it to remain secret.	194	11.6	63	3.8	1411	84.6
If you had TB, others would think less of you.	302	18.1	164	9.8	1202	72.1
If you had TB, you would be ashamed or embarrassed.	250	14.9	79	4.7	1339	80.3
If you had TB, others would avoid you.	403	24.2	205	12.3	1060	63.6
If you had TB, you would be asked to stay away from a social group.	251	15.1	191	11.5	1226	73.5
If you had TB, you would not disclose even to a confidant	164	9.8	91	5.5	1413	84.7
If you had TB, you would think less of yourself.	273	16.4	108	6.5	1287	77.2
If you had TB, others would think less of your family.	109	6.5	43	2.6	1516	90.9

A total of nine items were used to assess stigma and they had high internal consistency (Cronbach’s alpha = 0.98)

#### Families of TB patients

Among family members of TB patients, 514 (63.1%) reported that they could get TB, and 558 (68.5%) responded that they would cope with it if they did. Of the respondents, 685 (84.0%) reported that they would like to talk to medical personnel if they had TB. Some family members (8.9%, or 73) reported that they would not disclose their status even to a confidant (Additional file 1: Table S2).

Among family members, 637 (78.2%) mentioned that they knew people who had TB, and 637 (78.2%) reported that they would feel compassion and want to help them. Slightly more than half (432, or 53%) of the respondents mentioned that the community supported and helped TB patients. Some of the respondents - 205 (25.2%) and 176 (21.6%) -indicated that the community avoided and rejected them, respectively (Additional file 1: Table S1). One hundred and fifty-two (18.6%) and 134 (16%) respondents reported that others would avoid them and think less of them if they had TB, respectively.

#### TB patients

Most TB patients, 82.5% (679) reported that they would not disclose having TB to a confidant, while 107 (13%) would not disclose. Two hundred and forty-seven (30%) and 234 (28.5%) reported that they would not find a job or would lose their job, respectively (Additional file 1: Table S3). Of 380 who had no partner, 15.8% reported that they would have a problem in finding a spouse even after their cure. Of 443 who had a partner, 46.5% reported that their partner would refuse to have sex with them. Of 361 participants who had children, 80.1% of them reported that being a TB patient is a problem for their children (Additional file 1: Table S3).

### Stigma score and factors associated with stigma

#### General population

The mean stigma score was 18.6 (range: 9–45). Of the respondents, 645 (38.7%) had high stigma scores. A high stigma score was inversely correlated with educational status, region, and a high knowledge score. Addis Ababa had the lowest stigma score. Oromia had 10 times higher stigma compared to Addis Ababa (AOR: 0.1 [95% CI: 0.06–0.17]). Compared to those who could not read and write, those who were educated above secondary school had a 42% lower mean stigma score (AOR: 0.58 [95%CI: 0.39–0.87]). Respondents who had high knowledge scores had a lower 38% odds of having a high stigma score (AOR: 0.62 [95% CI: 0.49–0.78]) (Table 4).

**Table 4:** Factors associated with stigma towards tuberculosis in the general population

Variables		Stigma High	Stigma Low	COR (95% CI)	AOR (95%CI)
		# (%)	# (%)		
Gender	Male	293 (37.4)	490 (62.6)	0.91 (0.74–1.1)	NA
	Female	352 (39.8)	533 (60.2)	1	
Education	Not able to read and write	220 (46.5)	253 (53.5)	1	1
	Read and write only	48 (40.7)	70 (59.3)	0.79(0.52–1.19)	0.9(.57–1.38)
	Primary	180 (37.0)	306 (63.0)	0.68 (0.52–0.88)	0.84(.63–1.11)
	Secondary	143 (36.8)	246 (63.2)	0.67 (0.51–0.88)	0.92 (0.68–1.25)
	Above secondary	54 (26.7)	148 (73.3)	0.42 (0.29–0.6)	0.58 (0.39–0.87)^a^
Wealth	Lowest	122 (39.9)	184 (60.1)	1.53 (1.11–2.11)	0.83 (0.55–1.25)
	Second	146 (45.3)	176 (54.7)	1.91 (1.4–2.62)	0.97 (0.65–1.43)
	Third	137 (40.7)	200 (59.3)	1.58 (1.15–2.16)	0.88 (0.61–1.28)
	Fourth	132 (38.2)	214 (61.8)	1.42 (1.04–1.95)	0.85 (0.6–1.2)
	Highest	108 (30.3)	249 (69.7)	1	1
Setting	Rural	314 (42.5)	425 (57.5)	1.33 (1.1–1.63)	0.95 (0.75–1.21)
	Urban	331 (35.6)	598 (64.4)	1	1
Knowledge score	High	280 (33.5)	555 (66.5)	0.65 (0.53–0.79)	0.62 (0.49–0.78)^a^
	Low	365 (43.8)	468 (56.2)	1	1
Region	Oromia	192 (58.4)	137 (41.6)	1	1
	Amhara	121 (36.1)	214 (63.9)	0.4 (0.3–0.55)	0.35 (0.25–0.49)^a^
	SNNP	81 (25.5)	237 (74.5)	0.24 (0.18–0.34)	0.23 (0.16–0.32)^a^
	Tigray	95 (53.7)	82 (46.3)	0.83 (0.57–1.19)	0.8 (0.55–1.17)
	Benshangul Gumuz	43 (48.3)	46 (51.7)	0.67 (0.42–1.07)	0.61 (0.37–0.98)^a^
	Gambella	19 (23.2)	63 (76.8)	0.22 (0.12–0.38)	0.22 (0.12–0.38)^a^
	Addis Ababa	22 (12.7)	151 (87.3)	0.1 (0.06–0.17)	0.1 (0.06–0.17)^a^
	Dire Dawa	35 (43.2)	46 (56.8)	0.54 (0.33–0.89)	0.54 (0.33–0.91)^a^
	Harari	37 (44.0)	47 (56.0)	0.56 (0.35–0.91)	0.54 (0.32–0.88)^a^

^a^Statistically significant. The study participants were grouped as having high and low stigma score using the mean stigma score as a cut-off point

#### Families of TB patients

The mean stigma score was 20.46 (range: 10–46). Of the family members, 310 (38.0%) had high stigma scores. A high stigma score was associated with educational status and region. Respondents who had completed primary school (AOR: 0.6: 95% CI: 0.39–0.93), secondary school (AOR: 0.52; 95% CI: 0.32–0.84), and above secondary school education (AOR: 0.37: 95% CI: 0.23–0.61) were less likely to have high stigma scores compared to those who could not read and write. High stigma scores were observed in Oromia Region compared to other regions of Ethiopia (Additional file 1: Table S4).

#### TB patients

The mean stigma score was 21.31 (range: 9–45). Of the 844 TB patients, 356 (43.3%) had high stigma scores. A high stigma score was associated with educational status, wealth status, and region. Oromia Region had the highest and Addis Ababa had the least mean stigma scores. Compared to those who could not read and write, those with secondary school education had a 39% lower odds of having high stigma scores. TB patients in the first (AOR: 1.93: 95%CI 1.05–3.57) and third quintiles (AOR: 1.81: 95%CI: 1.08–3.05) had stigma scores twice as high as those in the highest wealth quintile (Additional file 1: Table S5).

Of the TB patients, 75.5% felt that family members were supportive. Most of the patients (75.3%) perceived that their utensils were separated. Close to half of TB patients (45.9%) feared reduced family income due to their condition, while 37% felt increased sadness, 32.5% felt the threat of losing their jobs, 25.4% mentioned that people behaved differently toward them, and 15.3% felt isolated within the family. Some (9.3%) felt avoided by family members (Table 5). Three-quarters (75.7%) of TB patients reported that they felt compassion for and a desire to help other TB patients, 7% reported no particular feeling, and 17.2% reported that they would stay away because of fear of re-infection (Additional file 1: Table S3).

**Table 5:** Perception of TB patients regarding their relationships and livelihood

Variables *N* = 823	Agree		Indifferent		Disagree	
	#	%	#	%	#	%
Family members are cooperative towards me	621	75.5	33	4.0	169	20.6
Utensils are separated for me	620	75.3	27	3.3	176	21.4
I have fear of reduction of family income	378	45.9	75	9.1	370	44.9
Threat of loss of job/wages	267	32.5	84	10.2	472	57.4
Most people behave differently	209	25.4	67	8.1	547	66.5
Feel isolated within the family	126	15.3	49	6.0	648	78.8
Family members avoid me	76	9.3	36	4.4	711	86.4
I have increased sadness	305	37.1	79	9.6	439	53.4

### Socioeconomic consequences of TB on the patients

Of the 844 TB patients, 64.9% reported that nothing had happened after they developed TB. However, 8.9% TB patients lost their jobs, 21% encountered a reduction in income, 1.5% divorced, and 3.4% interrupted school.

### Qualitative findings

The community felt that TB is a serious disease and treatment takes a long time, and they feared acquiring TB. People were afraid to share utensils with TB patients and sit near a TB patient who has a cough. Participants mentioned that TB patients are isolated or discriminated as against because TB is infectious and a communicable disease.

*“…TB patients have difficulty of getting houses to rent because of the fear of transmission of TB to people who live in the same compound…” (Addis Ababa, Female, FGD).*

*“My husband’s family stigmatized me a lot. Since they knew that I am a TB patient, they didn’t sleep in our house. They sleep outdoors. They are not also willing to eat with me. ...Before I was infected with TB, our social life with other people was great. The social life of DD community is well known. But after they knew that I am a TB patient, only one of my neighbors sometimes comes to visit me. In order not to come to my house frequently, she used to say I am tired and I have a lot of work to do at home” (Dire Dawa, Female TB patient).*

*“…acquiring TB may be considered as a curse…” (Addis Ababa, Female, FGD).*

Some participants reported that the extent of stigmatization and isolation of TB patients has significantly decreased these days because people know that it is curable. TB patients are not prohibited from obtaining services in the community due to their illness.

*“We should not discriminate [against] TB patients, rather we should help them with diets, proper care and anything else they require…” (Southern Nations, Nationalities, and Peoples’ Region, Male, FGD).*

Family members reported that they supported TB patients by providing nutritious food, arranging a separate bedroom, accompanying them when they go to collect drugs from the health centers/health posts, and allowing them to have adequate rest.

## Discussion

We report high fear of TB and stigma in the general population of Ethiopia, which are reflected by the community’s perception of TB patients and self-stigmatization by patients. Stigma is associated with educational status, level of awareness about TB, and wealth status, and its levels varied across regions.

Stigma related to TB is a perceived and/or internalized attitude of a community or families toward TB patients due to social norms. The poor, women, ethnic minorities, migrants, and refugees were reported to be highly affected by TB-related stigma and its consequences, including isolation, lack of support, and loss of employment, depending on the cultural context and level of awareness in the community [18, 19, 25, 26].

Community perceptions about TB can positively or negatively affect the capacity of the community to offer support to TB patients and the effectiveness of TB programs [18]. In settings where understanding and caring exist, the capacity of TB patients to seek care, adhere to treatment, and receive support [27]. Perceptions are shaped by knowledge about the disease, capacity to seek care, and factors affecting this capacity. Unfortunately, despite the existence of free and decentralized TB services, the attitudes, perceptions, and reactions of the community toward TB patients have affected service delivery and could result in delayed care seeking and affect treatment outcomes [5, 28]. TB remains a highly stigmatized disease, mainly because of its association with HIV and misconceptions about its transmission. This is confirmed by qualitative findings that TB patients were denied housing and sometimes the disease was considered a curse. In such communities, the capacity to access care is compromised and patients tend to remain at home rather than seek care when they develop symptoms.

As in our study, high self-disclosure to family members was reported from Nigeria. However, they believed that TB is an embarrassment to the family and did not share utensils or beds [29]. This could be due to TB-associated stigma within the community and associated consequences.

Perceptions about the disease also affect patients’ capacity to cope with it. In our study, 60% reported that they could cope with TB. The patients think less of themselves, however, and experience problems related to social engagement, employment, marriage, feelings of sadness, depression. and self-stigmatization [19, 30]. A study from Zambia found that stigma results in low self-esteem, affects disclosure capacity, and has social consequences for patients and their children, [15] findings that merit implementation of stigma reduction interventions.

Urban areas have higher knowledge about TB, which is reflected by lower stigma. However, in areas with high HIV prevalence, the opposite scenario existed, due to HIV-related stigma [8]. In other contexts, high knowledge scores were not paralleled by low stigma, which requires further study.

Stigma shapes the disclosure of TB status within a community. From the qualitative part of the study, we learned TB is sometimes considered a curse, which makes it more difficult for patients and households to disclose it to the community. Therefore, TB patients mainly disclosed their TB status to family members [31]. However, disclosure was affected by patients’ perception that they would not be stigmatized. Patients’ trust in the family and the community played a crucial role in supporting disclosure. In our study, the majority of study participants reported that they would seek public health facilities and disclose their symptoms to doctors. This could be due to low HIV-associated TB in rural communities, where patients dare to seek care and share information with health workers.

Patients affected by TB expressed stigma within the household, as reported from the FGDs in the qualitative part of the study. They indicated that the family members were not willing to share utensils or eat with them. More than the stigma from the community and families, internalized stigma by TB patients (self-stigmatization) plays an important role in care seeking and social engagement [10]. The fact that TB patients expect rejection by the community if they are known to have TB requires intervention to ensure that patients are accepted and supported when seeking care. This could be done through the decentralization of services, better health education about TB, and community support to patients. Engaging community HEWs and Ethiopia’s Health Development Army that reaches households is crucial to enhance the level of community support for patients. This is reflected in the qualitative part of the study, which underscores the importance of supporting TB patients in the community.

Ethiopia’s high TB burden may be due in part to TB-related stigma and perceptions about TB and sociocultural factors, as another study from Ethiopia has found [18]. The NTP needs to develop a stigma reduction strategy to reduce this barrier to seeking care. Stigma is associated with wealth, educational status, setting (rural/urban), and knowledge that TB is a preventable and curable disease, so tailored interventions are needed to the reach cases in the community missed as a result of stigma [19].

### Limitations of the study

This is the first national study about TB-related stigma in Ethiopia. However, cultural and socioeconomic conditions might have affected the understanding and expression of stigma in the communities. The interviewers speak the local language, created conducive environment and encouraged better communication by giving opportunities to compensate for this.

## Conclusions

TB-related stigma remains a challenge to TB prevention and control in Ethiopia. Therefore, tailored stigma reduction interventions are needed to increase community awareness about TB, improve health-seeking behavior, and promote support for TB patients in their households and the community.

## Supplementary information

**Additional file 1: Table S1.** Attitude and stigma related to TB among families of TB patients. **Table S2.** Responses of families of TB patients to TB stigma related questions. **Table S3.** Responses of TB patients to TB stigma related questions. **Table S4.** Factors associated with stigma towards tuberculosis in the families of TB patients. **Table S5.** Factors associated with stigma towards tuberculosis among TB patients.

## Data Availability

The datasets used and/or analysed during the current study available from the corresponding author on reasonable request.

## Abbreviations

DOTS: Directly observed treatment, short course
FGD: Focus group discussion
HEW: Health extension worker
HIV: Human immunodeficiency virus
IDI: In-depth interview
NTP: National Tuberculosis Programme
TB: Tuberculosis
WHO: World Health Organization
