# Supplementary material for: Stigma matters in ending tuberculosis: Nationwide survey of stigma in Ethiopia
Source: BMC Public Health. 2020 Feb 6;20:190. doi: 10.1186/s12889-019-7915-6 (PMC7006204; doi:10.1186/s12889-019-7915-6)
Supplement: Supplementary file 1 — Additional file 1: Table S1. Attitude and stigma related to TB among families of TB patients. Table S2. Responses of families of TB patients to TB stigma related questions. Table S3. Responses of TB patients to TB stigma related questions. Table S4. Factors associated with stigma towards tuberculosis in the families of TB patients. Table S5. Factors associated with stigma towards tuberculosis among TB patients. [file 12889_2019_7915_MOESM1_ESM.docx]

**Additional file 1**

**Table S1. Attitude and stigma related to TB among families of TB patients**

| **Variables** N=815 | | **#** | **%** |
| --- | --- | --- | --- |
| Do you think you can get TB | | 514 | 63.1 |
| Reaction if you were found out that you have TB (n=815) | Cope with it | 558 | 68.5 |
|  | Fear | 254 | 31.2 |
|  | Surprise | 37 | 4.5 |
|  | Shame | 17 | 2.1 |
|  | Embarrassment | 15 | 1.8 |
|  | Sadness or hopelessness | 37 | 4.5 |
|  | Other | 2 | 0.2 |
| Who would you talk to about your illness if you had TB? (n=815) | Doctor/other medical worker | 685 | 84.0 |
|  | Spouse | 180 | 22.1 |
|  | Parent | 240 | 29.4 |
|  | Children | 76 | 9.3 |
|  | Other family member | 204 | 25.0 |
|  | Close friend | 105 | 12.9 |
|  | No one | 4 | 0.5 |
|  | Others | 4 | 0.5 |
| What would you do if you thought you had symptoms of TB? (n=815) | Go to public health facility | 792 | 97.2 |
|  | Go to private health facility | 96 | 11.8 |
|  | Go to pharmacy | 25 | 3.1 |
|  | Go to spiritual/traditional healer | 7 | 0.9 |
|  | Pursue other self-treatment options | 2 | 0.2 |
|  | Others | 1 | 0.1 |
|  | Don’t know | 4 | 0.5 |
| If you would not go to health facility, what is the reason? (n=4) | Not sure where to go | 2 | - |
|  | Cost | 1 | - |
|  | Don’t want to find out something is really wrong | 1 | - |
| If you had symptoms of TB, at what point would you go to the health facility? (n=815) | Immediately | 484 | 59.4 |
|  | In few days | 160 | 19.6 |
|  | One to two weeks | 103 | 12.6 |
|  | After two weeks | 66 | 8.1 |
|  | I will not go to health facility | 2 | 0.2 |
| How expensive do you think TB diagnosis and treatment? (n=815) | It is free of charge | 560 | 68.7 |
|  | It is reasonably priced | 95 | 11.7 |
|  | It is moderately expensive | 36 | 4.4 |
|  | It is very expensive | 36 | 4.4 |
|  | Don’t know | 88 | 10.8 |
| Know people who have/had TB (n=815) | | 750 | 92.0 |
| Statement closest to your feeling about people with TB (n=815) | I feel compassion and desire to help | 637 | 78.2 |
|  | I feel compassion but tend to stay away from these people | 77 | 9.4 |
|  | It is their problem and don’t want to get TB by trying to help them | 46 | 5.6 |
|  | I fear them because they may infect me | 22 | 2.7 |
|  | I have no particular feeling | 33 | 4.0 |
| How is TB patient usually regarded/treated in your community? (n=815) | Most people reject him/her | 176 | 21.6 |
|  | Most people are friendly, but they generally try to avoid him/her | 205 | 25.2 |
|  | The community mostly supports and helps him/her | 432 | 53.0 |
|  | I don’t have the experience | 2 | 0.2 |

**Table S2. Responses of families of TB patients to TB stigma related questions**

| **Stigma related questions (N=815)** | **Agree** | | **Indifferent** | | **Disagree** | |
| --- | --- | --- | --- | --- | --- | --- |
|  | **#** | **%** | **#** | **%** | **#** | **%** |
| The fact that your family member has TB should be kept secret from your neighbors/community | 105 | 12.8 | 33 | 4.0 | 677 | 83.0 |
| If you got TB, you would want it to remain secret. | 105 | 12.8 | 33 | 4.0 | 677 | 83.0 |
| It is shameful to have a family member with TB | 93 | 11.4 | 36 | 4.4 | 686 | 84.2 |
| If you had TB, others would think less of you. | 134 | 16.4 | 80 | 9.8 | 601 | 73.7 |
| If you had TB, you would be ashamed or embarrassed. | 119 | 14.6 | 38 | 4.7 | 658 | 80.7 |
| If you had TB, others would avoid you. | 152 | 18.6 | 99 | 12.1 | 564 | 69.2 |
| If you had TB, you would be asked to stay away from a social group. | 108 | 13.3 | 88 | 10.8 | 619 | 76.0 |
| If you had TB, you would not disclose even to a confidant | 73 | 8.9 | 49 | 6.0 | 693 | 85.0 |
| If you had TB, you would think less of yourself. | 124 | 15.2 | 45 | 5.5 | 646 | 79.3 |
| If you had TB, others would think less of your family. | 22 | 2.7 | 13 | 1.6 | 780 | 95.7 |

^A total of ten items were used to assess stigma and they had high internal consistency (Cronbach’s alpha=0.9)^

**Table S3. Responses of TB patients to TB stigma related questions**

| **Stigma related questions (N=823)** | **Agree** | | **Indifferent** | | **Disagree** | |
| --- | --- | --- | --- | --- | --- | --- |
|  | **# (%)** | **%** | **#** | **%** | **#** | **%** |
| Being a TB patient, others would think less of you | 221 | 26.9 | 51 | 6.2 | 551 | 67.0 |
| Being a TB patient, you would be ashamed/embarrassed | 220 | 26.8 | 30 | 3.6 | 573 | 69.6 |
| Being a TB patient, other would avoid you | 202 | 24.6 | 54 | 6.6 | 567 | 68.9 |
| Being a TB patient, you would be asked to stay away from a social group | 150 | 18.2 | 67 | 8.1 | 606 | 73.7 |
| Being a TB patient, you would not disclose even to a confidant | 107 | 13.0 | 37 | 4.5 | 679 | 82.5 |
| Being a TB patient, you would think less of yourself | 195 | 23.7 | 54 | 6.6 | 574 | 69.7 |
| I Being a TB patient, other would think less of your family | 136 | 16.5 | 48 | 5.8 | 639 | 77.6 |
| Being a TB patient, you would be less likely to find a job | 247 | 30.0 | 92 | 11.2 | 484 | 58.8 |
| Being a TB patient, you would lose your job | 234 | 28.5 | 95 | 11.5 | 494 | 60.0 |

^A total of nine items were used to assess stigma and they had high internal consistency (Cronbach’s alpha=0.88)^

**Table S4. Factors associated with stigma towards tuberculosis in the families of TB patients.**

| **Variables** | | **Stigma High** | **Stigma Low** | **COR (95% CI)** | **AOR(95%CI)** |
| --- | --- | --- | --- | --- | --- |
|  |  | **# (%)** | **# (%)** |  |  |
| Gender | Male | 139(35.3) | 255(64.7) | 0.8(0.6-1.06) | 0.82(0.59-1.16) |
|  | Female | 171(40.6) | 250(59.4) | 1 | 1 |
| Education | Not able to read and write | 137(50.9) | 132(49.1) | 1 | 1 |
|  | Read and write only | 19(37.3)) | 32(62.7) | 0.57(0.31-1.06) | 0.53(0.26-1.07) |
|  | Primary | 79(35.6) | 143(64.4) | 0.53(0.37-0.77) | 0.6(0.39-0.93)* |
|  | Secondary | 47(27.3) | 125(72.7) | 0.36(0.24-0.55) | 0.52(0.32-0.84)* |
|  | Above secondary | 28(27.7) | 73(72.3) | 0.37(0.23-0.61) | 0.52(0.29-0.94)* |
| Wealth | Lowest | 67(44.1) | 85(55.9) | 2.73(1.68-4.43) | 1.44(0.74-2.78) |
|  | Second | 81(48.8) | 85(51.2) | 3.3(2.05-5.31) | 1.97(1.1-3.53)* |
|  | Third | 70(44.0) | 89(56.0) | 2.72(1.68-4.4) | 1.94(1.1-3.39)* |
|  | Fourth | 55(31.8) | 118(68.2) | 1.61(0.99-2.62) | 1.4(0.82-2.41) |
|  | Highest | 37(22.4) | 128(77.6) | 1 | 1 |
| Setting | Rural | 141(47.2) | 158(52.8) | 1.83(1.37-2.45) | 1.3(0.87-1.93) |
|  | Urban | 169(32.8) | 347(67.2) | 1 | 1 |
| Knowledge score | High | 150(36.6) | 260(63.4) | 0.91(0.67-1.23) | 0.97(0.69-1.37) |
|  | Low | 117(34.4) | 223(65.6) | 1 | 1 |
| Region | Oromia | 98(60.5) | 64(39.5) | 1 | 1 |
|  | Amhara | 56(33.1) | 113(66.9) | 0.32(0.21-0.51) | 0.52(0.3-0.9)* |
|  | SNNP | 58(35.4) | 106(64.6) | 0.36(0.23-0.56) | 0.5(0.3-0.84)* |
|  | Tigray | 36(42.9) | 48(57.1) | 0.49(0.29-0.84) | 0.85(0.45-1.58) |
|  | Benshangul Gumuz | 20(46.5) | 23(53.5) | 0.57(0.29-1.12) | 0.83(0.39-1.76) |
|  | Gambella | 5(20.0) | 20(80.0) | 0.16(0.06-0.46) | 0.22(0.07-0.65)* |
|  | Addis Ababa | 6(7.1) | 78(92.9) | 0.05(0.02-0.12) | 0.1(0.04-0.25)* |
|  | Dire Dawa | 17(40.5) | 25(59.5) | 0.44(0.22-0.89) | 1.06(0.48-2.33) |
|  | Harari | 14(33.3) | 28(66.7) | 0.33(0.16-0.67) | 0.54(0.24-1.2) |

^*Statistically significant; The study participants were grouped as having high and low stigma score using the mean stigma score as a cut-off point.^

**Table S5. Factors associated with stigma towards tuberculosis among TB patients**

| **Variables** | | **Stigma High** | **Stigma Low** | **COR (95% CI)** | **AOR (95%CI)** |
| --- | --- | --- | --- | --- | --- |
|  |  | **# (%)** | **# (%)** |  |  |
| Gender | Male | 203(42.3) | 277(57.7) | 0.91(0.69-1.2) | NA |
|  | Female | 153(44.6) | 190(55.4) | 1 |  |
| Education | Not able to read and write | 149(52.5) | 135(47.5) | 1 | 1 |
|  | Read and write only | 15(39.5) | 23(60.5) | 0.51(0.3-1.18) | 0.67(0.31-1.44) |
|  | Primary | 111(43.7) | 143(56.3) | 0.7(0.5-0.99) | 0.85(0.58-1.25) |
|  | Secondary | 51(31.3) | 112(68.7) | 0.41(0.28-0.62) | 0.61(0.38-0.97)* |
|  | Above secondary | 30(35.7) | 54(64.3) | 0.5(0.3-0.83) | 0.63(0.35-1.13) |
| Wealth | Lowest | 81(51.3) | 77(48.7) | 2.22(1.42-3.49) | 1.93(1.05-3.57)* |
|  | Second | 75(45.7) | 89(54.3) | 1.78(1.14-2.79) | 1.1(0.62-1.95) |
|  | Third | 97(56.1) | 76(43.9) | 2.7(1.73-4.2) | 1.81(1.08-3.05)* |
|  | Fourth | 50(30.7) | 113(69.3) | 0.94(0.59-1.49) | 0.75(0.45-1.25) |
|  | Highest | 53(32.1) | 112(67.9) | 1 | 1 |
| Setting | Rural | 169(49.7) | 171(50.3) | 1.56(1.18-2.07) | 1.02(0.7-1.47) |
|  | Urban | 187(38.7) | 296(61.3) | 1 | 1 |
| Knowledge score | High | 166(42.0) | 229(58.0) | 0.91(0.69-1.2) | 0.73(0.52-1.03) |
|  | Low | 190(44.4) | 238(55.6) | 1 | 1 |
| Region | Oromia | 120(71.4) | 48(28.6) | 1 | 1 |
|  | Amhara | 76(44.7) | 94(55.3) | 0.32(0.21-0.51) | 0.32(0.19-0.53)* |
|  | SNNP | 45(27.6) | 118(72.4) | 0.15(0.09-0.25) | 0.12(0.07-0.2)* |
|  | Tigray | 44(51.2) | 42(48.8) | 0.42(0.24-0.72) | 0.42(0.24-0.75)* |
|  | Benshangul Gumuz | 20(47.6) | 22(52.4) | 0.36(0.18-0.73) | 0.3(0.14-0.62)* |
|  | Gambella | 5(17.9) | 23(82.1) | 0.09(0.03-0.24) | 0.06(0.02-0.18)* |
|  | Addis Ababa | 11(13.4) | 71(86.6) | 0.06(0.03-0.13) | 0.08(0.04-0.18)* |
|  | Dire Dawa | 18(42.9) | 24(57.1) | 0.3(0.15-0.6) | 0.35(0.17-0.76)* |
|  | Harari | 17(40.5) | 25(59.5) | 0.27(0.14-0.55) | 0.29(0.14-0.62)* |

^*Statistically significant; The study participants were grouped as having high and low stigma score using the mean stigma score as a cut-off point.^
